# Outbreaks Associated with Untreated Recreational Water — California, Maine, and Minnesota, 2018–2019

**DOI:** 10.15585/mmwr.mm6925a3

**Published:** 2020-06-26

**Authors:** Kayla L. Vanden Esschert, Mia C. Mattioli, Elizabeth D. Hilborn, Virginia A. Roberts, Alexander T. Yu, Katherine Lamba, Gena Arzaga, Matthew Zahn, Zachary Marsh, Stephen M. Combes, Emer S. Smith, Trisha J. Robinson, Stephanie R. Gretsch, Joseph P. Laco, Mary E. Wikswo, Allison D. Miller, Danielle M. Tack, Timothy J. Wade, Michele C. Hlavsa

**Affiliations:** ^1^Division of Foodborne, Waterborne, and Environmental Diseases, National Center for Emerging and Zoonotic Infectious Diseases, CDC; ^2^Oak Ridge Institute for Science and Education, Oak Ridge, Tennessee; ^3^Environmental Protection Agency; Research Triangle Park, North Carolina; ^4^California Department of Public Health; ^5^Orange County Health Care Agency, Santa Ana, California; ^6^Maine Center for Disease Control and Prevention; ^7^University of Southern Maine, Portland, Maine; ^8^Minnesota Department of Health; ^9^Division of Environmental Health Science and Practice, National Center for Environmental Health, CDC; ^10^Division of Viral Diseases, National Center for Immunization and Respiratory Diseases, CDC; ^11^Eagle Medical Services, LLC, Atlanta, Georgia.

Outbreaks associated with fresh or marine (i.e., untreated) recreational water can be caused by pathogens or chemicals, including toxins. Voluntary reporting of these outbreaks to CDC’s National Outbreak Reporting System (NORS) began in 2009. NORS data for 2009–2017 are finalized, and data for 2018–2019 are provisional. During 2009–2019 (as of May 13, 2020), public health officials from 31 states voluntarily reported 119 untreated recreational water–associated outbreaks, resulting at least 5,240 cases; 103 of the outbreaks (87%) started during June–August. Among the 119 outbreaks, 88 (74%) had confirmed etiologies. The leading etiologies were enteric pathogens: norovirus (19 [22%] outbreaks; 1,858 cases); Shiga toxin–producing *Escherichia coli* (STEC) (19 [22%]; 240), *Cryptosporidium* (17 [19%]; 237), and *Shigella* (14 [16%]; 713). This report highlights three examples of outbreaks that occurred during 2018–2019, were caused by leading etiologies (*Shigella*, norovirus, or STEC), and demonstrate the wide geographic distribution of such outbreaks across the United States. Detection and investigation of untreated recreational water–associated outbreaks are challenging, and the sources of these outbreaks often are not identified. Tools for controlling and preventing transmission of enteric pathogens through untreated recreational water include epidemiologic investigations, regular monitoring of water quality (i.e., testing for fecal indicator bacteria), microbial source tracking, and health policy and communications (e.g., observing beach closure signs and not swimming while ill with diarrhea).

## California

On July 22, 2019, the California Department of Public Health was notified of three cases of shigellosis in persons who reported playing in the Santa Ana River, a waterway spanning 100 miles through southern California. The department identified this exposure in other shigellosis cases and, in total, identified 24 cases with closely related isolates (within 0–2 alleles by core-genome multilocus sequence typing) of *Shigella sonnei*. Among 19 ill persons for whom epidemiologic data were available, 16 reported that during July 6–August 5 they played in a swim area in a shallow portion of the river where water quality was not regularly monitored. Two of the 16 ill persons also reported swallowing river water. No other common risk factors were identified. The median age of these 16 ill persons was 7 years (range = 1–20 years); seven were female. Two of 15 ill persons for whom clinical data were available were hospitalized; none died. Date of symptom onset ranged from July 6 through August 7. In response to the outbreak, local public health officials closed public access to the swim area during August 8–15. Surface water samples were collected upstream, downstream, and at the swim area and tested for *E. coli,* a bacterial indicator of fecal contamination. The concentration of *E. coli* ranged from 350 through 1,600 most probable number/100 mL at these sites.* Investigation into possible sources of fecal contamination upstream and at the swim area did not definitively identify an outbreak source. No additional cases were identified after public access to the swim area was reopened on August 15.

## Maine

On July 6, 2018, the Maine Center for Disease Control and Prevention received a report that multiple persons were ill with gastrointestinal symptoms after visiting Woods Pond Beach in Bridgton, Maine. Town officials in Bridgton closed the public beach during July 6–10. The agency used social media to identify persons who visited the pond during July 1–6, interviewed 34 heads of household, and completed surveys for 148 household members. A total of 139 persons reported visiting the pond during this period, 97 (70%) of whom reported illness. Among these 97 ill persons, 41 (42%) were male; among the 95 ill persons for whom age data were available, the median age was 12 years (range = 1–73 years). The median incubation period was 38 hours (range = 8–139 hours); the median symptom duration, reported for 91 cases, was 24 hours (range = 3–96 hours). Vomiting was reported by 78 (80%) of 97 ill persons. Visitors who reported swallowing pond water or going under water (a potential marker for swallowing water) were approximately three times more likely to be ill than were those who did not (relative risk = 3.19; 95% confidence interval [CI] = 1.69–6.05). Two of the stool specimens collected from four ill persons tested positive for norovirus genogroup I. Based on these test results and the reported symptomology, norovirus was thought to be the outbreak etiology. The source of water contamination was undetermined. No additional cases were reported after the beach reopened to swimmers on July 11.

## Minnesota

On August 13, 2019, Minnesota Department of Health (MDH) epidemiologists identified three cases of STEC infection in persons who reported swimming at a public lake. Illness onset occurred during August 2–4. MDH notified park and recreation board officials of the cases on August 13 and advised them to close the lake to swimmers. MDH used social media to distribute a survey and identified 69 total cases, including four laboratory-confirmed STEC O145:H28 infections with closely related isolates (within 0–2 single nucleotide polymorphisms by whole genome sequencing). Dates of symptom onset ranged from July 18 through August 16. The median age of ill persons was 29 years (range = 1–65 years); 55 (80%) were female. Among the 24 (35%) ill persons who visited the beach only once, exposure dates ranged from July 16 through August 11. The two factors significantly associated with illness were swallowing lake water (odds ratio = 3.80; 95% CI = 1.17–12.38) and age ≤10 years (odds ratio = 2.90; 95% CI = 1.57–5.35). No hospitalizations or cases of hemolytic uremic syndrome were reported. The beach was monitored weekly for *E. coli* throughout the summer, but no test results exceeded Minnesota’s recreational water criteria during April–October.^†^ No evidence of a point source of fecal contamination was identified; however, 15 visitors and four lifeguards reported continuing to swim or work in the lake while ill. No additional cases were reported after the beach reopened to swimmers on September 5.

## Discussion

*Shigella*, norovirus, STEC, and other enteric pathogens can be transmitted when persons ingest untreated recreational water contaminated with feces or vomit. Swimmers can contaminate water in untreated recreational water venues (e.g., lakes, oceans, and rivers) if they have a fecal or vomit incident in the water. Enteric pathogens can also be introduced into untreated recreational water venues by stormwater runoff and sewage system overflows and discharges. Other potential sources of fecal contamination and enteric pathogens include leaks from septic or municipal wastewater systems, dumped boating waste, and animal waste in or near swim areas.

Whereas the detection of *Shigella* and norovirus in untreated recreational water is indicative of human contamination, the detection of STEC does not necessarily indicate human contamination. Because *E. coli* and enterococci are part of the normal intestinal flora of humans and other animals, beach managers monitor levels of these bacteria as indicators of fecal contamination as recommended by the Environmental Protection Agency’s 2012 recreational water quality criteria (*1*). Monitoring is conducted to detect changes in fecal contamination of water and not to indicate the presence of pathogens (*2*–*4*). For this reason, fecal indicator data alone cannot implicate the water as the route of outbreak exposure or identify the source of water contamination. This is particularly problematic for certain pathogenic strains of *E. coli*, such as *E. coli* O157:H7, which can persist in the sediment and be resuspended in the water but is not detected by most generic *E. coli* water tests.

In the outbreaks described in this report, the sources of contamination of the recreational waters were not definitively identified. Molecularly based microbial source tracking methods can be used to identify the host genus contributing to fecal contamination detected in water, which can inform more targeted environmental investigations and control measures (*5*). For example, identifying the host genus (e.g., horses) can help inform and optimize efforts to mitigate exposure (e.g., redesigning horse trails near untreated recreational water venues) to prevent outbreaks. Investigations into environmental influences include, but are not limited to, sanitary inspection of septic systems, identification of agricultural animal waste runoff or discharge, monitoring of wildlife activity in public areas, and identification of improper disposal of solid waste. 

Multiple factors could hinder detection and investigation of outbreaks associated with untreated recreational water venues. First, persons often travel >100 miles to swim in lakes, oceans, and rivers (*6*). If swimmers become ill after returning to homes in multiple public health jurisdictions, identifying an outbreak can be difficult. Second, not all jurisdictions include questions about exposure to recreational water in their investigations of cases of illness caused by enteric pathogens. Third, issues with response activities (e.g., collection of water samples and decision-making about closures) might arise among agencies within the same jurisdiction (e.g., public health and natural resources agencies) or among jurisdictions if the outbreak source (i.e., untreated recreational water venue) is in multiple jurisdictions.

In addition to monitoring the level of fecal indicator bacteria at beaches, beach managers can promote healthy swimming by establishing policies that allow lifeguards to perform alternate duties that do not require them to enter the water if they are ill with diarrhea. This is equivalent to CDC recommendations for operators of public treated recreational water venues (e.g., swimming pools)^§^ (*7*). Creating a workplace environment where employees feel comfortable disclosing that they are ill with diarrhea without fearing potential loss of wages or even work is important to the success of such policies. Because of the multiple potential sources of fecal contamination, beach managers and public health officials should educate swimmers and parents of young swimmers about steps they can take to minimize risk of infection from enteric pathogens (https://www.cdc.gov/healthywater/swimming/oceans-lakes-rivers/visiting-oceans-lakes-rivers.html). These healthy swimming steps include observing beach closure signs or water quality advisories because of elevated levels of fecal indicator bacteria, not swimming in water made cloudier by heavy rain, not swimming while ill with diarrhea, not swallowing the water, and keeping sand out of mouths. In addition, for the 2020 summer swim season, CDC has released coronavirus disease 2019 (COVID-19) prevention considerations for beach managers (https://www.cdc.gov/coronavirus/2019-ncov/community/parks-rec/public-beaches.html).

**Summary**

**What is already known about this topic?**

Untreated recreational water–associated outbreaks can be caused by pathogens or chemicals, including toxins, in freshwater (e.g., lakes) or marine water (e.g., oceans).

**What is added by this report?**

This report highlights examples of untreated recreational water–associated outbreaks that occurred during 2018 or 2019, were caused by *Shigella* (California), norovirus (Maine), or Shiga toxin–producing *Escherichia coli* (Minnesota), the leading causes of such outbreaks, and demonstrate the wide geographic distribution of such outbreaks.

**What are the implications for public health practice?**

Swimmers should observe beach closure signs and water quality advisories, not swim in water made cloudier by heavy rain, not swim while ill with diarrhea, not swallow recreational water, and keep sand out of their mouths.

## Footnotes

* Most probable number is a method used to estimate the concentration of viable bacteria in water. All samples exceeded the Environmental Protection Agency (EPA)–recommended Beach Action Values of 190–235 colony forming units (CFU)/100mL for freshwater. Beach Action Values are EPA’s suggested “do not exceed” value for beach advisory purposes.

† Minnesota recreational water criteria for freshwater call for a monthly geometric mean concentration of <126 CFU *E. coli*/100 mL water. For culturable *E. coli*, EPA criteria are a geometric mean concentration of 126 CFU/100mL and statistical threshold value of 410 CFU/100mL in freshwater.

§ CDC’s 2018 Model Aquatic Health Code (https://www.cdc.gov/mahc/pdf/2018-MAHC-Code-Clean-508.pdf) element 6.3.4.7.1 states “Supervisors shall not permit employees who are ill with diarrhea to enter the water or perform in a qualified lifeguard role.”
